# Feasibility of using a smartphone app to assess early signs, basic symptoms and psychotic symptoms over six months: A preliminary report

**DOI:** 10.1016/j.schres.2019.04.003

**Published:** 2019-06

**Authors:** Emily Eisner, Sandra Bucci, Natalie Berry, Richard Emsley, Christine Barrowclough, Richard James Drake

**Affiliations:** a University of Manchester, Division of Psychology and Mental Health, Zochonis Buildi ng (2^nd^ Floor), Brunswick Street, Manchester M13 9L, United Kingdom; b Greater Manchester Mental Health NHS Foundation Trust, Bury New Road, Prestwich, Manchester, Greater Manchester M25 3BL, United Kingdom; c Department of Biostatistics and Health Informatics, King's College London, Institute of Psychiatry, De Crespigny Park, Denmark Hill, London SE5 8AF, United Kingdom; d University of Manchester, Division of Psychology and Mental Health, Jean McFarlane Building (3^rd^ Floor), Manchester M13 9L, United Kingdom

**Keywords:** Schizophrenia, Psychosis, Relapse, mHealth, Telemedicine, Smartphone

## Abstract

**Background:**

Psychosis relapses are common, have profound adverse consequences for patients, and are costly to health services. ‘Early signs’ have been used to predict relapse, in the hope of prevention or mitigation, with moderate sensitivity and specificity. We investigated the feasibility and validity of adding ‘basic symptoms’ to conventional early signs and monitoring these using a smartphone app.

**Methods:**

Individuals (*n* = 18) experiencing a relapse within the past year were asked to use a smartphone app (‘ExPRESS’) weekly for six months to report early signs, basic symptoms and psychotic symptoms. Above-threshold increases in app-reported psychotic symptoms prompted a telephone interview (PANSS positive items) to assess relapse.

**Results:**

Participants completed 65% app assessments and 58% telephone interviews. App items showed high concurrent validity with researcher-rated psychotic symptoms and basic symptoms over six months. There was excellent agreement between telephone call and face-to-face assessed psychotic symptoms. The primary relapse definition, based on telephone assessment and casenotes, compared well with a casenote-only definition but had better specificity. Mixed-effects models provided preliminary evidence of concurrent and predictive validity: early signs and basic symptoms were associated with most app-assessed psychotic symptom variables the same week and with a number of psychotic symptoms variables three weeks later; adding basic symptoms to early signs improved model fit in most of these cases.

**Conclusions:**

This is the first study to test a smartphone app for monitoring early signs and basic symptoms as putative relapse predictors. It demonstrates that weekly app-based monitoring is feasible, valid and acceptable over six months.

## 1 Introduction

Psychosis relapses are associated with worse outcomes by almost every measure (Almond et al., 2004; Andrew et al., 2012; Appleby, 1992; Birchwood et al., 2000; Gumley and Schwannauer, 2006; Iqbal et al., 2000; Maclean, 2008; Wiersma et al., 1998; Wu et al., 2005). Relapse signatures, idiosyncratic combinations of warning signs, have been used to predict relapse in the hope of prevention or mitigation but have only moderate sensitivity and specificity (Eisner et al., 2013). To improve predictive power, we investigated adding basic symptoms (Schultze-Lutter et al., 2007a) to pre-existing putative predictors (conventional early signs) (Birchwood et al., 1989) and using a smartphone application (‘app’) to facilitate prompt identification of these.

Incorporating both basic symptoms and conventional early signs of relapse into personalized relapse signatures will likely achieve better relapse prediction than has previously been demonstrated (Eisner et al., 2013). Basic symptoms are subtle, subjective changes in individuals' experiences of themselves (e.g. mild cognitive problems) and the world around them (e.g. more vivid colors) which predict first episodes of psychosis (Fusar-Poli et al., 2012; Schultze-Lutter et al., 2007b). There is preliminary evidence that basic symptoms also predict relapses of psychosis (Bechdolf et al., 2002; Eisner et al., 2018; Gaebel and Riesbeck, 2014) but there are no comprehensive, prospective studies examining this. A well-powered, methodologically sound, prospective study to establish whether basic symptoms predict relapses of psychosis is needed. We tested the feasibility of carrying out such a study using a smartphone app, ExPRESS (Experiences of Psychosis Relapse: Early Subjective Signs) (Eisner et al., 2019), which collects weekly assessments of early signs, basic symptoms and psychotic symptoms.

Existing early signs studies have typically used pen and paper questionnaires (Birchwood et al., 1989; Gaebel et al., 1993; Gaebel and Riesbeck, 2007, Gaebel and Riesbeck, 2014; Gleeson et al., 2005; Gumley et al., 2015; Hirsch and Jolley, 1989; Jørgensen, 1998; Malla and Norman, 1994; Marder et al., 1991; Marder et al., 1994; Subotnik and Neuchterlein, 1988; Tait et al., 2002; Tarrier et al., 1991) or text message systems (Spaniel et al., 2018; Spaniel et al., 2007; Spaniel et al., 2008) to examine the predictive value of conventional early signs of relapse. Compared to these methods, smartphone apps have a number of advantages: apps can be accessed at the individual's convenience (Ben-Zeev et al., 2013), decreasing participant burden and increasing ecological validity (Bucci et al., 2018); apps can automatically supply surveys and securely upload responses; finally, apps are more acceptable to individuals with psychosis than text message systems (Ainsworth et al., 2013).

A number of symptom monitoring apps prospectively assessing symptom course have been tested (Ainsworth et al., 2013; Barnett et al., 2018; Ben-Zeev et al., 2014; Ben-Zeev et al., 2017; Ben-Zeev et al., 2018; Bucci et al., 2018; Kumar et al., 2018; Meyer et al., 2018; Niendam et al., 2018; Palmier-Claus et al., 2012). Most monitor individuals' current mental state rather than aiming to elicit symptoms predictive of relapse. Two assessed symptoms overlapping somewhat with conventional early signs (e.g. anxiety, confusion), but did not measure relapse (Kumar et al., 2018; Niendam et al., 2018). A small pilot study (Barnett et al., 2018) collected both self-reported early signs via an app and relapse as an outcome, but the focus of the published paper was mainly on the predictive value of passively collected data, with limited details of early signs data reported. Although a number of studies have reported good correspondence between passively collected data and outcomes (Barnett et al., 2018; Meyer et al., 2018; Wang et al., 2016; Wang et al., 2017), this was not the focus of the current study. Instead, we aimed to further refine the predictive value of app-based monitoring by adding basic symptoms to conventional early signs as putative relapse predictors.

The current study tested the methodology for a planned large-scale study examining whether basic symptoms are valuable relapse predictors. There were four specific aims:

i) to explore the feasibility of using a smartphone app (ExPRESS) for weekly monitoring of early signs, basic symptoms, psychotic symptoms and relapse over six months;
ii) to assess the concurrent and preliminary predictive validity of using personalized relapse signatures integrating basic symptoms and conventional early signs;
iii) to examine the validity of an operational definition of relapse using a combination of smartphone app assessment, verbal telephone assessment and casenote examination;
iv) to examine the acceptability of the study procedures.

## 2 Materials and methods

### 2.1 Study design

This study consisted of three phases. First, cross-sectional assessments characterized the sample and checked eligibility for the next phase. Second, eligible participants used ExPRESS for six months and received telephone calls from the researcher (prospective, longitudinal phase). Finally, after six months the acceptability of the study procedures was explored using qualitative interviews. The study was carried out in accordance with the Declaration of Helsinki (World Medical Association, 2013), ethical approval was obtained from Greater Manchester West Research Ethics Committee (14/NW/1471) and the study was registered (ClinicalTrials.gov: NCT03558529).

### 2.2 Participants

Participants were recruited from three Mental Health Trusts in North-West England between June 2015 and June 2016. Inclusion criteria were: schizophrenia spectrum diagnosis (Diagnostic and Statistical Manual of Mental Disorders, 4th Edition; DSM-IV) (Sheehan et al., 1998); ≥1 acute psychotic episode in the past year (admission to crisis team or hospital; or exacerbation of psychotic symptoms lasting ≥2 weeks and leading to a change in management), or ≥2 episodes in the past 2 years, including index episode; currently prescribed antipsychotic medication; age over 18 years; fixed abode; fluent in English; sufficiently stable to take part (able to complete screening assessment); no current alcohol or drug dependence (Structured Clinical Interview for DSM-IV) (First et al., 1996); informed consent. To progress beyond baseline assessment, individuals must have reported basic symptoms which began or increased prior to a recent episode of psychosis.

### 2.3 ExPRESS app

App design is detailed elsewhere (Eisner et al., 2019), with screenshots provided in Supplementary Fig. 1. Briefly, ExPRESS is an android smartphone app which prompts participants once a week to answer a personalized set of questions regarding psychotic symptoms (PANSS positive items) (Ainsworth et al., 2013; Kay et al., 1987; Palmier-Claus et al., 2012), mood symptoms (Calgary Depression Scale) (Addington et al., 1993), basic symptoms (Basic Symptoms Checklist, BSC) (Eisner et al., 2019) and early signs of relapse (Early Signs Scale, ESS) (Birchwood et al., 1989) within the past week. Participants have a 24-h window each week to respond to the question set. Responses are uploaded automatically to a secure server, accessible to the research team via a password protected web interface. Weekly self-reports were deemed sufficiently frequent to allow early signs to be meaningfully detected but not so frequent as to overburden participants, since beta-test participants considered weekly app use acceptable but would not have wanted it to be more frequent (Eisner et al., 2019).

### 2.4 Procedure

#### 2.4.1 Baseline assessments

**Fig. 1:**
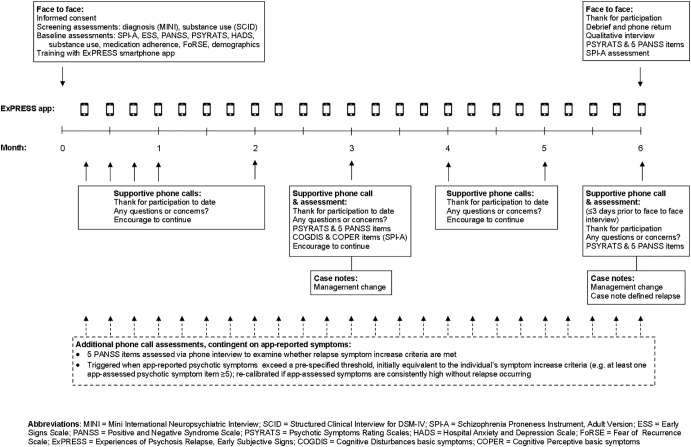
Overview of assessments.

An overview of all baseline and follow-up assessments is provided in Fig. 1. At baseline, the Schizophrenia Proneness Instrument Adult Version interview (SPI-A) (Schultze-Lutter et al., 2007a) identified whether participants had experienced basic symptoms beginning or increasing prior to their most recent psychotic episode. Early signs (ESS) (Birchwood et al., 1989) were assessed for the same period.

Psychotic symptoms and mood symptoms during the previous week were assessed using the PANSS (Kay et al., 1987), PSYRATS (Psychotic Symptom Rating Scales) (Haddock et al., 1999) and Hospital Anxiety and Depression Scale (Zigmond and Snaith, 1983). Substance use was assessed using a 4-point scale (Tarrier et al., 2006), medication adherence using a 7-point scale (Kemp et al., 1996) and cognitions related to imminent psychosis relapse using the Fear of Recurrence Scale (FoRSE) (Gumley et al., 2015). Demographic information was gathered using a standard questionnaire. Assessors were trained and supervised by senior colleagues. Mean intra-class correlations (ICC) compared to gold standard PANSS ratings were excellent (positive = 0.92; negative = 0.83; general = 0.89; total = 0.92).

#### 2.4.2 Six month longitudinal app-use phase

Using SPI-A and ESS assessments, the researcher and participant defined a ‘relapse signature’ combining early signs and basic symptoms. Items from the participant's relapse signature were entered into the ExPRESS app so that individuals could monitor a personalized set of early signs. Participants were trained on ExPRESS and asked to use it weekly for 6 months or until relapse, whichever was sooner. Those owning an android smartphone used their own phones and the remaining participants used a study phone. The latter received weekly text messages to their own phone reminding them to use ExPRESS.

Participants were telephoned by the researcher (weekly for four weeks; monthly thereafter) to encourage participation and troubleshoot any difficulties with app use. During the 3-month telephone call, the researcher assessed the PSYRATS, five PANSS positive items (delusions, hallucinations, suspiciousness, grandiosity, conceptual disorganization), and a subset of SPI-A items (Cognitive Disturbances (COGDIS) and Cognitive-Perceptive (COPER)) (Schultze-Lutter et al., 2007a). This was to screen for additional delusions or overlooked relapses and to check app item validity.

The primary relapse definition (Wunderink et al., 2007) (Supplementary Fig. 2) required a symptom increase for ≥1 week resulting in a management change (casenote-reported medication change or increased observation by the clinical team, including admission). Symptom increase criteria, assessed via PANSS telephone interview (five items: delusions, hallucinations, suspiciousness, grandiosity, conceptual disorganization), were: for remitted individuals, an increase to ≥4 or an increase of ≥2 points (whichever was higher) on any item; for non-remitted individuals with all baseline PANSS positive items <5, at least one item ≥5; for non-remitted individuals with ≥1 baseline item ≥5, an increase to ≥4 or an increase of ≥1 point (whichever was higher) on any item. Individuals' remission status was initially determined from baseline PANSS using standard remission severity criteria (Andreasen et al., 2005). To identify participants remitting during the app-use phase, we modified these criteria to accommodate app content: participants scoring ≤3 for two consecutive weeks on all app-assessed psychotic symptom items (delusions, hallucinations, grandiosity, suspiciousness; scaled the same as corresponding PANSS items) were classified as remitted.

PANSS telephone interviews were triggered when app-reported psychotic symptoms exceeded a pre-specified threshold (initially equivalent to the symptom increase criteria). If symptom increase criteria were met, the telephone assessment was repeated one week later to check duration. If app-reported symptoms exceeded the threshold two consecutive weeks without any increase in symptoms detected during the resultant telephone call, the threshold for receiving a call was recalibrated (for each app-assessed symptom, the new threshold was one point above the average of the previous four app responses).

Two aspects of the above relapse assessment procedure were added seven months into the study. Firstly, the original definition did not account for remission status, with identical symptom increase criteria applied to non-remitted and remitted individuals. Secondly, the telephone call threshold recalibration was formerly absent, so participants with high residual symptoms were telephoned every week. Following these changes, four were deemed remitted at baseline, three as remitted during the app-use phase, four had their telephone call threshold recalibrated and five were unaffected.

The secondary relapse definition, assessed using casenotes alone, required a symptom increase for ≥1 week resulting in a management change. For each participant, the relapse start date was recorded, with verbatim extracts from casenotes describing changes in symptoms and management. The researcher conducting casenote screening was trained to protocol (Barrowclough et al., 2010); ratings on reliability cases showed perfect correspondence with gold standard assessors (relapse presence/absence kappa = 1.00; relapse start date ICC = 1.00).

#### 2.4.3 Qualitative interviews and final assessments

On longitudinal phase completion or dropout, face-to-face assessments (SPI-A; PANSS positive items) and qualitative interviews were conducted. The brief (average 6 min), audio-recorded qualitative interview topic guide included: reasons for participation; researcher support; telephone call acceptability and financial reimbursement; study highlights and lowlights. PANSS positive items were assessed via telephone within three days prior to face-to-face assessment for validity checks.

#### 2.4.4 Participant payment

Participants received: £10 per completed study phase; £10 monthly to cover phone credit (longitudinal participants); £5 per telephone interview (last 6 months of the study only; to test whether engagement increased).

### 2.5 Study outcomes and analysis

#### 2.5.1 Statistical analysis

Analyses were conducted in Stata (StataCorp, 2015), with bootstrapping where appropriate, and considered statistically significant at *p* < 0.05. App-assessed items scored on a 4-point scale were linearly transformed to a 7-point scale to aid comparison with other measures.

#### 2.5.2 ExPRESS weekly monitoring: feasibility

Feasibility was assessed using descriptive statistics summarizing: recruitment to baseline assessments, recruitment to longitudinal phase (proportion of baseline participants eligible and consenting), app engagement (percentage of assessments completed), timing of app responses across the 24-hour response window, proportion of standard study telephone calls and telephone call PANSS assessments completed, and proportion of study phones returned. The 6-month relapse rate was noted, to inform future power calculations. The pattern of app completion during the app-use phase was examined in a mixed-effects model with a random effect of participant and a fixed-effect of time. Effects of baseline variables on percentage app completion were examined using Spearman's correlation (continuous variables), Mann-Whitney or Kruskal-Wallis test (categorical variables).

#### 2.5.3 Relapse signatures (basic symptoms and early signs): validity

To measure concurrent validity, the number of app-reported basic symptoms during the first three and full six months of app use were compared to retrospective telephone (3 months) and face-to-face (6 months) researcher-rated SPI-A assessments using ICCs (two way mixed, absolute agreement, single measures). Preliminary predictive validity was assessed in two ways. Firstly, mixed-effects models were estimated, with app-assessed psychotic symptoms as the dependent variable, a fixed-effect of early signs and/or basic symptoms and a random effect of participant. Likelihood ratio tests explored whether adding basic symptoms to early signs improved model fit. Secondly, patterns of basic symptoms and early signs were examined graphically in the participants meeting full or partial relapse definitions.

#### 2.5.4 Relapse definition: validity

Validity was examined by comparing: 6-month relapse rates using primary and secondary relapse definitions (kappa); telephone interviews of PANSS items and the same items assessed face-to-face (two way mixed, absolute agreement, single measures ICC); researcher-rated symptoms and app-reported symptoms. For the latter comparison, the researcher-rated symptom variables came from a face-to-face interview, where available, or telephone interview. Spearman's correlation was calculated to aid comparison with previous studies (Palmier-Claus et al., 2012) but this does not account for the nested data structure. Therefore, mixed-effects models were constructed, with app-reported symptoms as the dependent variable, a fixed-effect of researcher-rated symptoms and a random effect of participant. The fixed-effect coefficient can be interpreted as the average change in app-reported symptoms for a 1-point change in researcher-rated symptoms.

#### 2.5.5 Study procedures: acceptability

We used the framework method (Gale et al., 2013) to analyze verbatim transcriptions of qualitative interviews. The research team developed the initial analytical framework using the topic guide and independently coded two of the first three transcripts each, updating the framework as necessary. The first author systematically coded the remaining transcripts and charted the data into a framework matrix to aid discussion and interpretation by the whole team.

## 3 Results

### 3.1 Sample characteristics

Demographic and clinical characteristics of longitudinal participants are shown in Table 1.

**Table 1:** Clinical and demographic characteristics of longitudinal phase sample (n = 18).

	Baseline variable	Association between baseline variable and percentage app use		
	Frequency (percentage) unless otherwise stated	Test statistic type	Test statistic value	*P* value
Diagnosis				
Schizophrenia	14 (77.8)	U	23.00	0.622
Schizoaffective	4 (22.2)			
PANSS subscales (mean, sd)				
Positive	15.4 (5.4)	ρ	−0.22	0.380
Negative	15.1 (4.7)	ρ	−0.09	0.725
General	31.0 (8.8)	ρ	−0.37	0.128
Total	61.8 (16.8)	ρ	−0.26	0.308
PSYRATS subscales (mean, sd)				
Delusions	6.6 (6.3)	ρ	−0.36	0.148
Hallucinations	17.4 (12.9)	ρ	−0.23	0.368
HADS subscales (mean, sd)				
Anxiety	8.9 (6.1)	ρ	−0.48	0.052
Depression	7.6 (6.3)	ρ	−0.56	0.015
FoRSE subscales (mean, sd)				
Fear of Relapse	14.5 (5.8)	ρ	−0.58	0.014
Intrusions	13.8 (5.6)	ρ	−0.21	0.419
Awareness	18.1 (6.1)	ρ	−0.34	0.163
Total	45.9 (14.7)	ρ	−0.40	0.128
Medication adherence (median, range)	6 (3, 7)	Χ^2^	4.57	0.188
Gender, n male	12 (66.7)	U	20.00	0.141
Age (mean, sd)	37.9 (9.9)	ρ	−0.14	0.570
Education				
Secondary	10 (55.6)	Χ^2^	0.20	0.914
Further	5 (27.8)			
Higher	3 (16.7)			
Employment				
Employed	2 (11.1)	Χ^2^	4.38	0.195
Voluntary work	1 (5.6)			
Retired	1 (5.6)			
Unemployed	14 (77.8)			
Ethnicity				
Asian or Asian British	1 (5.6)	Χ^2^	0.63	0.792
Black or Black British	2 (11.1)			
White British	15 (83.3)			
Marital status				
Single	14 (77.8)	Χ^2^	1.53	0.516
Married	2 (11.1)			
Separated	2 (11.1)			
Living arrangement				
Alone	12 (66.7)	Χ^2^	3.53	0.558
With family	4 (22.2)			
Supported accommodation	2 (11.1)			
Owns a smartphone	15 (83.3)	U	15.50	0.520
Used a study phone	13 (72.2)	U	26.50	0.580
Consent to give app data to clinician	15 (83.3)	U	13.00	0.278

Abbreviations: PANSS = Positive and Negative Syndrome Scale; PSYRATS = Psychotic Symptom Rating Scales; HADS = Hospital Anxiety and Depression Scale; FoRSE = Fear of Recurrence Scale;

Test statistics: U = Mann-Whitney *U* test statistic; ρ = Spearman's correlation coefficient; Χ^2^ = Kruskall Wallis test statistic.

### 3.2 ExPRESS weekly monitoring: feasibility

**Fig. 2:**
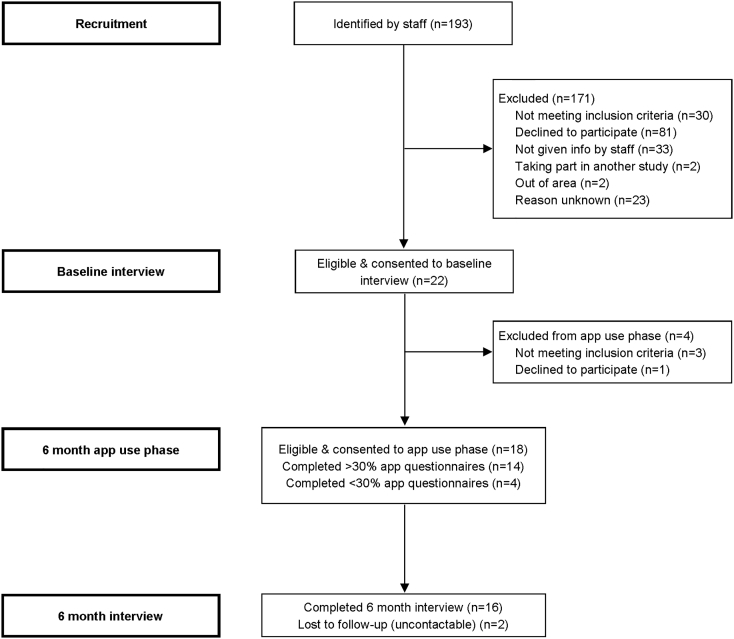
Consort Diagram.

Fig. 2 shows recruitment and retention. Only 11% (22/193) of individuals that clinical staff considered potentially suitable participated in baseline interviews. The main reasons for exclusion were that individuals were ineligible, declined to participate, or were not given study information by staff. Most baseline participants (18/22) were eligible for and consented to the longitudinal phase and engagement was high, with 78% completing ≥33% app assessments and 72% completing ≥50% app assessments. Most longitudinal participants (89%) completed a qualitative interview. Thirteen participants used ExPRESS on a study phone; all returned the phone in good condition except one who returned it to a clinician, who lost it.

**Fig. 3:**
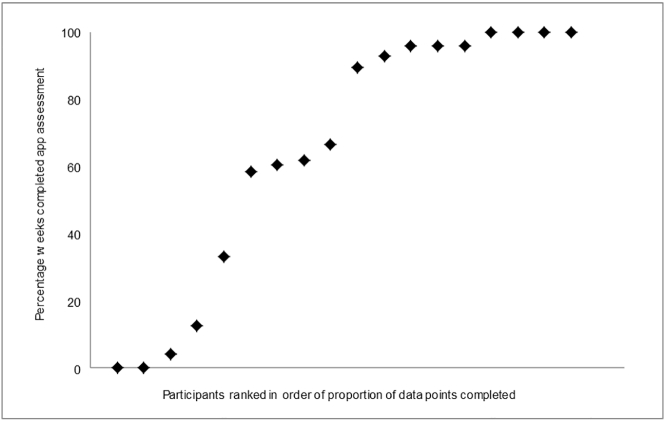
Distribution of app completion during the 6 month app-use period across the sample (*n* = 18).

Participants completed 65% app assessments and 65% supportive telephone calls. App-reported symptoms increased above the telephone interview threshold 31 times, of which 18 (58%) resulted in completed PANSS positive telephone interviews; participants were unavailable for the remaining 13 telephone interviews. The distribution of app completion across the sample is shown in Fig. 3. Three clusters are apparent: half the sample completed ≥90% of app assessments, four completed 60–70% and four completed <13% of assessments. The exception was unexpectedly abroad for several months, precluding app completion; while in the UK, their app completion was 60%.

Supplementary Fig. 3 shows app response timing across the 24-hour response window; most responses occurred in the first 12 h, with a substantial peak immediately after the initial alert (1.30 pm). Participants responded to fewer prompts as the study progressed (OR = 0.89 per week follow-up; *p* < 0.001; Supplementary Fig. 4). Percentage app completion was significantly and inversely correlated with baseline depression and fear of relapse, with baseline anxiety approaching significance and all other baseline variables non-significant (Table 1).

### 3.3 Relapse signatures (basic symptoms and early signs): validity

#### 3.3.1 Descriptive statistics

The mean number of basic symptoms reported at baseline as having begun or increased prior to a recent psychotic episode was 6.4 (sd = 4.3, range = 1–17); mean ESS score for this period was 84.4 (sd = 20.6, range = 41–121).

#### 3.3.2 Concurrent validity

ICCs comparing the number of app-reported and researcher-rated basic symptoms suggested poor agreement at 3 months (ICC = 0.37, *p* = 0.307, *n* = 8; telephone call COPER/COGDIS items) but excellent agreement at 6 months (ICC = 0.76, *p* < 0.001, *n* = 12; face-to-face full SPI-A).

In mixed-effects models (Table 3) both early signs and basic symptoms were significantly associated with all psychotic symptom measures, except grandiosity, at the same time point. Adding basic symptoms to early signs improved model fit in three cases.

#### 3.3.3 Prediction of psychotic symptoms (Table 3)

One week later: early signs significantly predicted suspiciousness; neither early signs nor basic symptoms predicted any other psychotic symptom variables.

Two weeks later: basic symptoms approached significance in predicting suspiciousness; no other predictions two weeks later were significant.

Three weeks later: early signs significantly predicted psychotic symptoms and hallucinations; basic symptoms significantly predicted psychotic symptoms and delusions, with hallucinations and suspiciousness approaching significance; adding basic symptoms to early signs improved model fit for psychotic symptoms and delusions, with suspiciousness approaching significance.

#### 3.3.4 Prediction of relapse

Graphs of app-assessed items for participants meeting full or partial relapse definitions (Supplementary Fig. 5) indicate greater variability over time in severity of app-assessed early signs and basic symptoms than app- or researcher-assessed psychotic symptoms. Since they respond more than psychotic symptoms to relapse triggers and treatment, this probably signifies greater sensitivity to underlying changes rather than more random noise.

### 3.4 Relapse definition: validity

Two participants met both primary and secondary relapse definitions, and one met the secondary definition alone, a substantial agreement (kappa = 0.76, *p* = 0.003). The relapse which met the secondary but not primary definition appeared to be a false positive (see Supplementary Textbox 1). Agreement between five PANSS positive items assessed during a telephone call and the same items assessed face-to-face was extremely high (Table 2), with ICCs ranging from 0.94 to 0.96 (p < 0.001). High agreement was also demonstrated for PSYRATS delusions (ICC = 0.97, p < 0.001) and hallucinations (ICC = 0.89, p < 0.001) subscales. The correlation between researcher-rated symptoms and app-reported symptoms was high, with Spearman's rho ranging from 0.80 to 0.87 (p < 0.001; Table 2). Fixed-effects coefficients from mixed-effects models ranged from 0.66 to 1.08 (*p* < 0.05), with all confidence intervals crossing 1.00.

**Table 2:** Comparison of three modes of assessing psychotic symptoms: face-to-face interview, telephone interview, self-report using app items.

	Face-to-face vs. phone call^a^		Researcher-rated^b^ vs. app-reported			
	ICC		Spearman's rho		Mixed effects model^c^	
	Coefficient	95% CI	Coefficient	95% CI	Coefficient	95% CI
Delusions	0.96⁎⁎	0.92, 1.01	0.80⁎⁎	0.66, 0.95	0.66⁎	0.14, 1.18
Conceptual disorganization	0.96⁎⁎	0.87, 1.05	–	–	–	–
Hallucinations	0.94⁎⁎	0.86, 1.03	0.87⁎⁎	0.77, 0.97	1.08⁎⁎	0.90, 1.26
Grandiosity	0.96⁎⁎	0.77, 1.16	0.84⁎⁎	0.69, 1.00	1.02⁎⁎	0.85, 1.19
Suspiciousness	0.96⁎⁎	0.91, 1.01	0.85⁎⁎	0.73, 0.97	0.94⁎	0.18, 1.68
PSYRATS^d^ delusions	0.97⁎⁎	0.91, 1.02	–	–	–	–
PSYRATS^d^ hallucinations	0.89⁎⁎	0.70, 1.09	–	–	–	–

⁎⁎ *p* < 0.001.

⁎ *p* < 0.05.

a Based on 14 or 15 cases depending on item.

b Face-to-face interview, where available, or telephone interview when no face-to-face interview available; based on 27–37 observations from 14 to 15 cases, depending on item.

c Mixed effects model with bootstrapping with app-reported symptoms as the dependent variable, a fixed effect of researcher-rated symptoms and a random effect of participant.

d Psychotic Symptom Rating Scales.

**Table 3:** Mixed effects models examining whether app-assessed conventional early signs and basic symptoms separately predict app-assessed psychotic symptoms and whether adding basic symptoms to conventional early signs improves model fit (likelihood-ratio test).

	Early signs				Basic symptoms				Likelihood-ratio test	
Prediction of:	Coefficient	SE	P value	Observations	Coefficient	SE	P value	Observations	LR Chi2	P value
Simultaneous psychosis										
Psychotic symptoms	0.435	0.059	**<0.001**	275	0.314	0.051	**<0.001**	275	12.20	**<0.001**
Hallucinations	0.556	0.076	**<0.001**	275	0.442	0.064	**<0.001**	275	17.49	**<0.001**
Delusions	0.347	0.076	**<0.001**	275	0.214	0.067	**0.002**	275	2.24	0.134
Suspiciousness	0.311	0.073	**<0.001**	274	0.288	0.075	**<0.001**	274	11.23	**<0.001**
Grandiosity	0.071	0.083	0.391	275	0.014	0.051	0.788	275	0.11	0.742
Psychosis 1 week later										
Psychotic symptoms	0.104	0.075	0.169	223	0.081	0.068	0.232	222	0.69	0.407
Hallucinations	0.122	0.091	0.180	223	0.116	0.088	0.189	222	1.14	0.286
Delusions	0.117	0.089	0.190	223	0.078	0.088	0.387	222	0.29	0.591
Suspiciousness	0.194	0.080	**0.016**	222	0.060	0.077	0.435	221	0.19	0.667
Grandiosity	−0.090	0.080	0.262	223	−0.107	0.071	0.131	222	1.26	0.263
Psychosis 2 weeks later										
Psychotic symptoms	0.028	0.086	0.744	209	0.084	0.067	0.211	208	2.39	0.122
Hallucinations	0.127	0.120	0.293	209	0.132	0.103	0.202	208	1.85	0.174
Delusions	−0.027	0.097	0.778	209	0.067	0.078	0.388	208	1.96	0.162
Suspiciousness	−0.023	0.084	0.789	208	0.121	0.068	0.075	207	6.47	**0.011**
Grandiosity	0.007	0.089	0.941	209	0.044	0.080	0.577	208	0.45	0.501
Psychosis 3 weeks later										
Psychotic symptoms	0.192	0.076	**0.011**	206	0.174	0.067	**0.009**	205	6.25	**0.012**
Hallucinations	0.283	0.094	**0.003**	206	0.162	0.091	0.075	205	1.63	0.201
Delusions	0.138	0.096	0.152	206	0.216	0.090	**0.017**	205	11.02	**<0.001**
Suspiciousness	0.056	0.078	0.472	205	0.110	0.058	0.056	204	3.55	0.060
Grandiosity	0.071	0.093	0.444	206	0.083	0.078	0.293	205	0.89	0.345

Bold values statistically significance at p < 0.05.

### 3.5 Study procedures: acceptability

We report the acceptability of study participation (Supplementary Table 1). A detailed analysis of ExPRESS's acceptability is reported elsewhere (Eisner et al., 2019). Participants gave various reasons for taking part, including altruism, wanting to increase knowledge about psychosis, curiosity about the research, feeling it might help them personally and previous positive research experiences. One individual, initially attracted by the financial reimbursement, became genuinely interested in the study. Several participants reported liking answering the questions, enjoying their novelty and finding them easy to answer even during difficult weeks. Others enjoyed the normalizing effect of ExPRESS, satisfaction of helping with research and increased understanding of their illness.

All participants found study telephone interviews acceptable and felt they received enough researcher support, with several mentioning that the reminder texts were helpful. Participants commented that the telephone calls were not intrusive and their frequency was appropriate; some found them encouraging and reassuring. Views about whether financial reimbursement for telephone interviews was needed were mixed. Although not specifically asked about the baseline interview, two participants mentioned finding it long and a little stressful initially; another commented that he found it acceptable.

## 4 Discussion

This study demonstrates the feasibility and acceptability of using an app for weekly monitoring of early signs, basic symptoms, psychotic symptoms and relapse over a six-month period, alongside telephone calls from a researcher. We show preliminary evidence of concurrent and predictive validity: early signs and basic symptoms were separately associated with most app-assessed psychotic symptom variables the same week and with a number of psychotic symptoms variables three weeks later, and adding basic symptoms to early signs improved model fit in most of these cases. App items showed high concurrent validity with researcher-rated psychotic symptoms and basic symptoms over six months. There was also excellent agreement between telephone call and face-to-face assessed psychotic symptoms. The primary definition of relapse, based on telephone interviews and casenotes, compared well with a casenote-only definition but had better specificity.

Since this study has the longest app-use phase of any to date reporting a symptom monitoring app in a sample with established psychosis, participants' app engagement is of key interest. Participants completed 65% app assessments, with 78% completing ≥33% assessments. Although lower than studies with a follow-up ≤2 months (Ainsworth et al., 2013; Ben-Zeev et al., 2014; Meyer et al., 2018; Palmier-Claus et al., 2012), this compares favorably to those with 3-month (Bucci et al., 2018) or 5-month (Kumar et al., 2018) follow-up periods. Unlike previous studies (Ainsworth et al., 2013; Ben-Zeev et al., 2014; Bucci et al., 2018; Kumar et al., 2018; Palmier-Claus et al., 2012), we used weekly (rather than daily) app assessments which appear to be better tolerated over longer follow-up periods. However, the only study with a longer follow-up (14 months) (Niendam et al., 2018) included daily app assessments and averaged 69% app completion in a clinical high risk and recent onset psychosis sample. Participants were paid per completed assessment which likely increased engagement. Their young age may also have engendered higher app completion since they are likely to be more familiar with smartphone apps than our older sample with established psychosis (Bonet et al., 2018). Although neither we, nor others (Meyer et al., 2018; Palmier-Claus et al., 2012), found an effect of age on app completion, our sample was possibly too small or had an insufficient age range to detect this.

Other predictors of app engagement have been examined but a consistent picture is yet to emerge (Killikelly et al., 2017). Studies have reported that higher positive (Meyer et al., 2018; Palmier-Claus et al., 2012), negative (Kumar et al., 2018; Meyer et al., 2018) or agitation/mania symptoms (Kumar et al., 2018) predicted lower app engagement, whereas the current study found a significant effect of depression and fear of relapse. Arguably, those who are more fearful of relapse and more depressed are at greater risk of relapse (Conley, 2009; Gumley et al., 2015), more likely to avoid help seeking (Gumley and Park, 2010) and thus more difficult for services to engage in treatment. If symptom monitoring apps become commonplace, clinicians must recognize that these may not suit everyone and take additional steps to engage these individuals. Interestingly, neither we, nor others (Kumar et al., 2018), found that using a study phone significantly reduced app engagement, despite qualitative feedback suggesting that participants prefer their own phones (Ainsworth et al., 2013; Eisner et al., 2019; Palmier-Claus et al., 2012). Return rate of study phones in the current study was excellent (92%), and comparable to previous studies (Biagianti et al., 2017; Granholm et al., 2012), contrasting with clinicians' fears that patients might lose/sell study phones (Berry et al., 2017).

This study provides a novel approach to operationally defining relapse by using a combination of telephone interviews and casenotes. While numerous relapse definitions exist, these rely on face-to-face assessments or solely on casenotes (Eisner et al., 2013; Falloon et al., 1983; Gleeson et al., 2010; Olivares et al., 2013). We suggest that remote relapse assessment is more easily integrated into participants' lives, decreasing both participant and researcher burden; this is likely to increase engagement and allow more frequent, long-term monitoring. Our operational relapse definition compared well with a casenote-only definition but had better specificity, since the latter generated a false positive. However, conversely, our operational definition may have generated false negatives; since the proportion of unanswered telephone calls was high (42%), some symptom increases may have been missed. As the first study comparing telephone and face-to-face interview PANSS items, our finding of extremely high agreement between these is encouraging but needs replicating in a larger sample. Nevertheless, this finding suggests that our definition may be comparable to other relapse definitions that use PANSS positive items.

We found a strong association between app-reported and researcher-rated psychotic symptoms, replicating previous findings (Niendam et al., 2018; Palmier-Claus et al., 2012) and suggesting that symptom monitoring apps are a valid means of assessing symptom course. Agreement between app- and researcher-assessed basic symptoms was also excellent over 6 months, improving upon previous self-report measures of basic symptoms which show poor concurrent validity (Mass et al., 1997; Michel et al., 2016). Although we found low agreement at 3-month assessment, key differences between the 3- and 6-month SPI-A assessments may explain this disparity: the 3-month researcher-rated assessment was conducted via telephone rather than face-to-face, with a smaller sample and only included a sub-set of SPI-A items.

There are a number of important limitations. Firstly, the sample was small, precluding a definitive examination of predictive validity: sensitivity and specificity could not be calculated, making it difficult to compare with other early signs studies. Nonetheless, mixed-effects models (using changes in psychotic symptoms as a proxy for relapse) and graphs of relapsers provided sufficient evidence of predictive validity to warrant further investigation. Secondly, the sample is unlikely to be representative, as the decline rate was high (42%), albeit comparable with other symptom monitoring app studies (Ainsworth et al., 2013: ~30% decline; Bucci et al., 2018: 30%; Ben-Zeev et al., 2014: 39%; Kumar et al., 2018: 44%; Niendam et al., 2018: 47%; Palmier-Claus et al., 2012: ~50%). Thirdly, researchers were not formally blind to participants' early signs or basic symptoms prior to conducting PANSS telephone interviews; in practice neither early signs nor basic symptoms were known but future studies should use an independent, formally blinded assessor. Fourthly, regarding the mixed-effects models, the dependent variable (psychotic symptoms) and predictors (early signs and basic symptoms) are related experiences which were self-reported by participants using the same method (an app), increasing the risk of spurious correlations arising from common method variance and expectancy effects. Fifthly, while baseline assessments were conducted by a single assessor, a second assessor carried out half the 6-month SPI-A assessments but inter-rater reliability was not evaluated. Finally, since this was a feasibility study, protocol changes were made during data collection. However, these changes refined the relapse definition and reduced the number of telephone interviews.

In conclusion, we found that weekly app-based assessment of symptoms was feasible, acceptable and valid over a six month period, offering support for a large-scale study using this methodology. More generally, these findings provide further evidence that symptom monitoring technology could be a valuable addition to routine mental health service delivery, since they closely correspond to researcher-rated assessments and are well-tolerated over an extended period of time.

## Conflict of interest

Sandra Bucci is a director of Affigo CIC, a not-for-profit social enterprise company spun out of the University of Manchester in December 2015 to enable access to social enterprise funding and to promote ClinTouch, a symptom-monitoring app, to the NHS and public sector.

## Contributors

All authors were involved in the design and ongoing management of the study, and contributed to drafts of this report. EE, the principal investigator, prepared the protocol, was responsible for the day-to-day running of the study, collected, analysed and interpreted the data and took a lead on writing the manuscript. SB and RD provided clinical and research supervision. RE advised on statistical aspects of the study design and analysis. NB provided maternity cover.

## Role of the funding source

The Medical Research Council did not play a role in the study design, data collection, data analysis, data interpretation, writing of the report or in the decision to submit the article for publication.

## Appendix A Supplementary data

Supplementary material Image 1

## References

[bb0005] Addington D., Addington J., Maticka-Tyndale E. (1993). Assessing depression in schizophrenia: the Calgary Depression Scale. Br J Psychiatry Suppl.

[bb0010] Ainsworth J., Palmier-Claus J., Machin M., Barrowclough C., Dunn G., Rogers A., Buchan I., Barkus E., Kapur S., Wykes T., Hopkins R.S., Lewis S. (2013). A comparison of two delivery modalities of a mobile phone-based assessment for serious mental illness: native smartphone application vs text-messaging only implementations. J. Med. Internet Res..

[bb0015] Almond S., Knapp M., Francois C., Toumi M., Traolach B. (2004). Relapse in schizophrenia: costs, clinical outcomes and quality of life. Br. J. Psychiatry.

[bb0020] Andreasen N.C., Carpenter W.T., Kane J.M., Lasser R.A., Marder S.R., Weinberger D.R. (2005). Remission in schizophrenia: proposed criteria and rationale for consensus. Am. J. Psychiatry.

[bb0025] Andrew A., Knapp M., McCrone P., Parsonage M., Trachtenberg M. (2012). Effective Interventions in Schizophrenia the Economic Case: A Report Prepared for the Schizophrenia Commission.

[bb0030] Appleby L. (1992). Suicide in psychiatric patients: risk and prevention. Br. J. Psychiatry.

[bb0035] Barnett I., Torous J., Staples P., Sandoval L., Keshavan M., Onnela J.P. (2018). Relapse prediction in schizophrenia through digital phenotyping: a pilot study. Neuropsychopharmacology.

[bb0040] Barrowclough C., Haddock G., Wykes T., Beardmore R., Conrod P., Craig T., Davies L., Dunn G., Eisner E., Lewis S., Moring J., Steel C., Tarrier N. (2010). Integrated motivational interviewing and cognitive behavioural therapy for people with psychosis and comorbid substance misuse: randomised controlled trial. BMJ.

[bb0045] Bechdolf A., Schultze-Lutter F., Klosterkötter J. (2002). Self-experienced vulnerability, prodromal symptoms and coping strategies preceding schizophrenic and depressive relapses. Eur Psychiatry.

[bb0050] Ben-Zeev D., Kaiser S.M., Brenner C.J., Begale M., Duffecy J., Mohr D.C. (2013). Development and usability testing of FOCUS: a smartphone system for self-management of schizophrenia. Psychiatr Rehabil J.

[bb0055] Ben-Zeev D., Brenner C.J., Begale M., Duffecy J., Mohr D.C., Mueser K.T. (2014). Feasibility, acceptability, and preliminary efficacy of a smartphone intervention for schizophrenia. Schizophr. Bull..

[bb0060] Ben-Zeev D., Brian R., Wang R., Wang W., Campbell A.T., Aung M.S.H., Merrill M., Tseng V.W.S., Choudhury T., Hauser M., Kane J.M., Scherer E.A. (2017). CrossCheck: integrating self-report, behavioral sensing, and smartphone use to identify digital indicators of psychotic relapse. Psychiatr Rehabil J.

[bb0065] Ben-Zeev D., Brian R.M., Jonathan G., Razzano L., Pashka N., Carpenter-Song E., Drake R.E., Scherer E.A. (2018). Mobile health (mHealth) versus clinic-based group intervention for people with serious mental illness: a randomized controlled trial. Psychiatr. Serv..

[bb0070] Berry N., Bucci S., Lobban F. (2017). Use of the internet and mobile phones for self-management of severe mental health problems: qualitative study of staff views. JMIR Ment Health.

[bb0075] Biagianti B., Hidalgo-Mazzei D., Meyer N. (2017). Developing digital interventions for people living with serious mental illness: perspectives from three mHealth studies. Evid Based Ment Health.

[bb0080] Birchwood M., Smith J., Macmillan F., Hogg B., Prasad R., Harvey C., Bering S. (1989). Predicting relapse in schizophrenia: the development and implementation of an early signs monitoring system using patients and families as observers, a preliminary investigation. Psychol. Med..

[bb0085] Birchwood M., Iqbal Z., Chadwick P., Trower P. (2000). Cognitive approach to depression and suicidal thinking in psychosis. 1. Ontogeny of post-psychotic depression. Br. J. Psychiatry.

[bb0090] Bonet L., Llacer B., Hernandez-Viadel M., Arce D., Blanquer I., Canete C., Escarti M., Gonzalez-Pinto A.M., Sanjuan J. (2018). Differences in the use and opinions about new eHealth technologies among patients with psychosis: structured questionnaire. JMIR Ment Health.

[bb0095] Bucci S., Barrowclough C., Ainsworth J., Machin M., Morris R., Berry K., Emsley R., Lewis S., Edge D., Buchan I., Haddock G. (2018). Actissist: proof-of-concept trial of a theory-driven digital intervention for psychosis. Schizophr. Bull..

[bb0100] Conley R.R. (2009). The burden of depressive symptoms in people with schizophrenia. Psychiatr Clin North Am.

[bb0105] Eisner E., Drake R., Barrowclough C. (2013). Assessing early signs of relapse in psychosis: review and future directions. Clin. Psychol. Rev..

[bb0110] Eisner E., Drake R., Lobban F., Bucci S., Emsley R., Barrowclough C. (2018). Comparing early signs and basic symptoms as methods for predicting psychotic relapse in clinical practice. Schizophr. Res..

[bb0115] Eisner E., Drake R.J., Berry N., Barrowclough C., Emsley R., Machin M., Bucci S. (2019). Development and long-term acceptability of ExPRESS, a mobile phone app to monitor basic symptoms and early signs of psychosis relapse. JMIR mHealth and uHealth.

[bb0120] Falloon I.R., Marshall G.N., Boyd J.L., Razani J., Wood-Siverio C. (1983). Relapse in schizophrenia: a review of the concept and its definitions. Psychol. Med..

[bb0125] First, M.B., Spitzer, R.L., M., G., Williams, J.B.W., 1996. Structured Clinical Interview for DSM-IV Axis I Disorders, Clinician Version (SCID-CV). American Psychiatric Press, Inc., Washington, D.C.

[bb0130] Fusar-Poli P., Bonoldi I., Yung A.R., Borgwardt S., Kempton M.J., Valmaggia L., Barale F., Caverzasi E., McGuire P. (2012). Predicting psychosis: meta-analysis of transition outcomes in individuals at high clinical risk. Arch. Gen. Psychiatry.

[bb0135] Gaebel W., Riesbeck M. (2007). Revisiting the relapse predictive validity of prodromal symptoms in schizophrenia. Schizophr. Res..

[bb0140] Gaebel W., Riesbeck M. (2014). Are there clinically useful predictors and early warning signs for pending relapse?. Schizophr. Res..

[bb0145] Gaebel W., Frick U., Kopcke W., Linden M., Muller P., Muller-Spahn F., Pietzcker A., Tegeler J. (1993). Early neuroleptic intervention in schizophrenia: are prodromal symptoms valid predictors of relapse?. Br. J. Psychiatry.

[bb0150] Gale N.K., Heath G., Cameron E., Rashid S., Redwood S. (2013). Using the framework method for the analysis of qualitative data in multi-disciplinary health research. BMC Med. Res. Methodol..

[bb0155] Gleeson J.F., Rawlings D., Jackson H.J., McGorry P.D. (2005). Early warning signs of relapse following a first episode of psychosis. Schizophr. Res..

[bb0160] Gleeson J.F., Alvarez-Jimenez M., Cotton S.M., Parker A.G., Hetrick S. (2010). A systematic review of relapse measurement in randomized controlled trials of relapse prevention in first-episode psychosis. Schizophr. Res..

[bb0165] Granholm E., Ben-Zeev D., Link P.C., Bradshaw K.R., Holden J.L. (2012). Mobile assessment and treatment for schizophrenia (MATS): a pilot trial of an interactive text-messaging intervention for medication adherence, socialization, and auditory hallucinations. Schizophr. Bull..

[bb0170] Gumley A., Park C., French P., Reed M., Smith J., Rayne M., Shiers D. (2010). Relapse prevention in early psychosis. Early Recovery in Psychosis: Promoting Recovery.

[bb0175] Gumley A., Schwannauer M. (2006). Staying Well after Psychosis: A Cognitive Interpersonal Approach to Recovery and Relapse Prevention.

[bb0180] Gumley A.I., MacBeth A., Reilly J.D., O'Grady M., White R.G., McLeod H., Schwannauer M., Power K.G. (2015). Fear of recurrence: results of a randomized trial of relapse detection in schizophrenia. Br J Clin Psychol.

[bb0185] Haddock G., McCarron J., Tarrier N., Faragher E.B. (1999). Scales to measure dimensions of hallucinations and delusions: the psychotic symptom rating scales (PSYRATS). Psychol. Med..

[bb0190] Hirsch S., Jolley A. (1989). The dysphoric syndrome in schizophrenia and its implications for relapse. Br. J. Psychiatry.

[bb0195] Iqbal Z., Birchwood M., Chadwick P., Trower P. (2000). Cognitive approach to depression and suicidal thinking in psychosis. 2. Testing the validity of a social ranking model. Br. J. Psychiatry.

[bb0200] Jørgensen (1998). Early signs of psychotic relapse in schizophrenia. Br. J. Psychiatry.

[bb0205] Kay S.R., Fiszbein A., Opler L.A. (1987). The positive and negative syndrome scale (PANSS) for schizophrenia. Schizophr. Bull..

[bb0210] Kemp R., Hayward P., Applewhaite G., Everitt B., David A. (1996). Compliance therapy in psychotic patients: randomised controlled trial. BMJ.

[bb0215] Killikelly C., He Z., Reeder C., Wykes T. (2017). Improving adherence to web-based and mobile technologies for people with psychosis: systematic review of new potential predictors of adherence. JMIR Mhealth Uhealth.

[bb0220] Kumar D., Tully L.M., Iosif A.M., Zakskorn L.N., Nye K.E., Zia A., Niendam T.A. (2018). A Mobile health platform for clinical monitoring in early psychosis: implementation in community-based outpatient early psychosis care. JMIR Ment Health.

[bb0225] Maclean C. (2008). An Interpretative Phenomenological Analysis of Service users' Perspectives and Experiences of Relapse in Psychosis.

[bb0230] Malla A.K., Norman R.M. (1994). Prodromal symptoms in schizophrenia. Br. J. Psychiatry.

[bb0235] Marder S., Mintz J., Van Putten T., Lebell M., Wirshing W., Johnston-Cronk K. (1991). Early prediction of relapse in schizophrenia: an application of receiver operating characteristic (ROC) methods. Psychopharmacol. Bull..

[bb0240] Marder S., Wirshing W., Van Putten T., Mintz J., McKenzie J., Johnston-Cronk K., Lebell M., Liberman R. (1994). Fluphenazine vs placebo supplementation for prodromal signs of relapse in schizophrenia. Arch. Gen. Psychiatry.

[bb0245] Mass R., Hitschfeld K., Wall E., Wagner H.B. (1997). Validität der Erfassung schizophrener Basissymptome. Nervenarzt.

[bb0250] Meyer N., Kerz M., Folarin A., Joyce D., Jackson R., Karr C., Dobson R., MacCabe J. (2018). Capturing rest-activity profiles in schizophrenia using wearable and mobile technologies: development, implementation, feasibility, and acceptability of a remote monitoring platform. JMIR Mhealth Uhealth.

[bb0255] Michel C., Kutschal C., Schimmelmann B., Schultze-Lutter F. (2016). Convergent and concurrent validity of the Frankfurt Complaint Questionnaire as a screener for psychosis risk. Journal of Risk Research.

[bb0260] Niendam T.A., Tully L.M., Iosif A.M., Kumar D., Nye K.E., Denton J.C., Zakskorn L.N., Fedechko T.L., Pierce K.M. (2018). Enhancing early psychosis treatment using smartphone technology: a longitudinal feasibility and validity study. J. Psychiatr. Res..

[bb0265] Olivares J.M., Sermon J., Hemels M., Schreiner A. (2013). Definitions and drivers of relapse in patients with schizophrenia: a systematic literature review. Ann. General Psychiatry.

[bb0270] Palmier-Claus J., Ainsworth J., Machin M., Barrowclough C., Dunn G., Barkus E., Rogers A., Wykes T., Kapur S., Buchan I., Salter E., Lewis S. (2012). The feasibility and validity of ambulatory self-report of psychotic symptoms using a smartphone software application. BMC Psychiatry.

[bb0275] Schultze-Lutter F., Addington J., Ruhrmann S., Klosterkötter J. (2007). Schizophrenia Proneness Instrument: Adult Version (SPI-A).

[bb0280] Schultze-Lutter F., Klosterkötter J., Picker H., Steinmeyer E.M., Ruhrmann S. (2007). Predicting first-episode psychosis by basic symptom criteria. Clin. Neuropsychiatry.

[bb0285] Sheehan D.V., Lecrubier Y., Sheehan K.H., Amorim P., Janavs J., Weiller E., Hergueta T., Baker R., Dunbar G.C. (1998). The Mini-International Neuropsychiatric Interview (M.I.N.I.): the development and validation of a structured diagnostic psychiatric interview for DSM-IV and ICD-10. J Clin Psychiatry.

[bb0290] Spaniel F., Novak T., Motlova L., Hrdlicka J., Hoschl C. (2007). Information technology aided relapse prevention program in schizophrenia (ITAREPS): reliability and validity of the early warning signs questionnaire. Psychiatrie.

[bb0295] Spaniel F., Vohlidka P., Hrdlicka J., Kozeny J., Novak T., Motlova L., Cermak J., Bednarik J., Novak D., Hoschl C. (2008). ITAREPS: information technology aided relapse prevention programme in schizophrenia. Schizophr. Res..

[bb0300] Spaniel F., Bakstein E., Anyz J., Hlinka J., Sieger T., Hrdlicka J., Gornerova N., Hoschl C. (2018). Relapse in schizophrenia: definitively not a bolt from the blue. Neurosci. Lett..

[bb0305] StataCorp (2015). Stata Statistical Software.

[bb0310] Subotnik K., Neuchterlein K. (1988). Prodromal signs and symptoms of schizophrenic relapse. J. Abnorm. Psychol..

[bb0315] Tait A., McNay L., Gumley A., O'Grady M. (2002). The development and implementation of an individualised early signs monitoring system in the prediction of relapse in schizophrenia. J. Ment. Health.

[bb0320] Tarrier N., Barrowclough C., Bamrah J.S. (1991). Prodromal signs of relapse in schizophrenia. Soc. Psychiatry Psychiatr. Epidemiol..

[bb0325] Tarrier N., Haddock G., Lewis S., Drake R., Gregg L., So C.T.G. (2006). Suicide behaviour over 18 months in recent onset schizophrenic patients: the effects of CBT. Schizophr. Res..

[bb0330] Wang R., Aung M.S.H., Abdullah S., Brian R., Campbell A.T., Choudhury T., Hauser M., Kane J., Merrill M., Scherer E.A., Tseng V.W.S., Ben-Zeev D. (2016). CrossCheck: Toward passive sensing and detection of mental health changes in people with schizophrenia. Proceedings of the 2016 ACM International Joint Conference on Pervasive Ubiquitous Computing.

[bb0335] Wang R., Wang W., Aung M.S.H., Ben-Zeev D., Brian R., Campbell A.T., Choudhury T., Hauser M., Kane J., Scherer E.A., Walsh M. (2017). Predicting symptom trajectories of schizophrenia using mobile sensing. Proceedings of the ACM on Interactive, Mobile, Wearable and Ubiquitous Technologies.

[bb0340] Wiersma D., Nienhuis F., Slooff C., Giel R. (1998). Natural course of schizophrenic disorders: a 15-year followup of a Dutch incidence cohort. Schizophr. Bull..

[bb0345] World Medical Association (2013). World medical association declaration of Helsinki: ethical principles for medical research involving human subjects. JAMA.

[bb0350] Wu E.Q., Birnbaum H.G., Shi L., Ball D.E., Kessler R.C., Moulis M., Aggarwal J. (2005). The economic burden of schizophrenia in the United States in 2002. J Clin Psychiatry.

[bb0355] Wunderink L., Nienhuis F.J., Sytema S., Slooff C.J., Knegtering R., Wiersma D. (2007). Guided discontinuation versus maintenance treatment in remitted first-episode psychosis: relapse rates and functional outcome. J Clin Psychiatry.

[bb0360] Zigmond A.S., Snaith R.P. (1983). The hospital anxiety and depression scale. Acta Psychiatr. Scand..

