# Supplementary material for: Feasibility of using a smartphone app to assess early signs, basic symptoms and psychotic symptoms over six months: A preliminary report
Source: Schizophr Res. 2019 Jun;208:105–13. doi: 10.1016/j.schres.2019.04.003 (PMC6551369; doi:10.1016/j.schres.2019.04.003)
Supplement: Supplementary file 1 — Supplementary material [file mmc1.pdf]

Schizophrenia Research

Supplementary material to:

Feasibility of using a smartphone app to assess early signs, basic symptoms and psychotic symptoms over six months: A preliminary report

Authors:

Emily Eisner [a]

Sandra Bucci [a, b]

Natalie Berry [a]

Richard Emsley [c]

Christine Barrowclough [a]

Richard James Drake [b, d]

[a] University of Manchester, Division of Psychology and Mental Health, Zochonis Building (2nd Floor), Brunswick Street, Manchester, M13 9L, United Kingdom

[b] Greater Manchester Mental Health NHS Foundation Trust, Bury New Road, Prestwich, Manchester, Greater Manchester, M25 3BL

[c] Department of Biostatistics and Health Informatics, King's College London, Institute of Psychiatry, De Crespigny Park, Denmark Hill, London SE5 8AF, United Kingdom

[d] University of Manchester, Division of Psychology and Mental Health, Jean McFarlane Building (3rd Floor), Manchester, M13 9L, United Kingdom

Corresponding author:

Emily Eisner

Address: 2nd Floor, Zochonis Building, Brunswick Street, Manchester. M13 9PL, UK

Email: emily.eisner@manchester.ac.uk

Supplementary Fig. 1: Screenshots of an example basic symptom item (left) and early signs item (right) displayed on the ExPRESS app

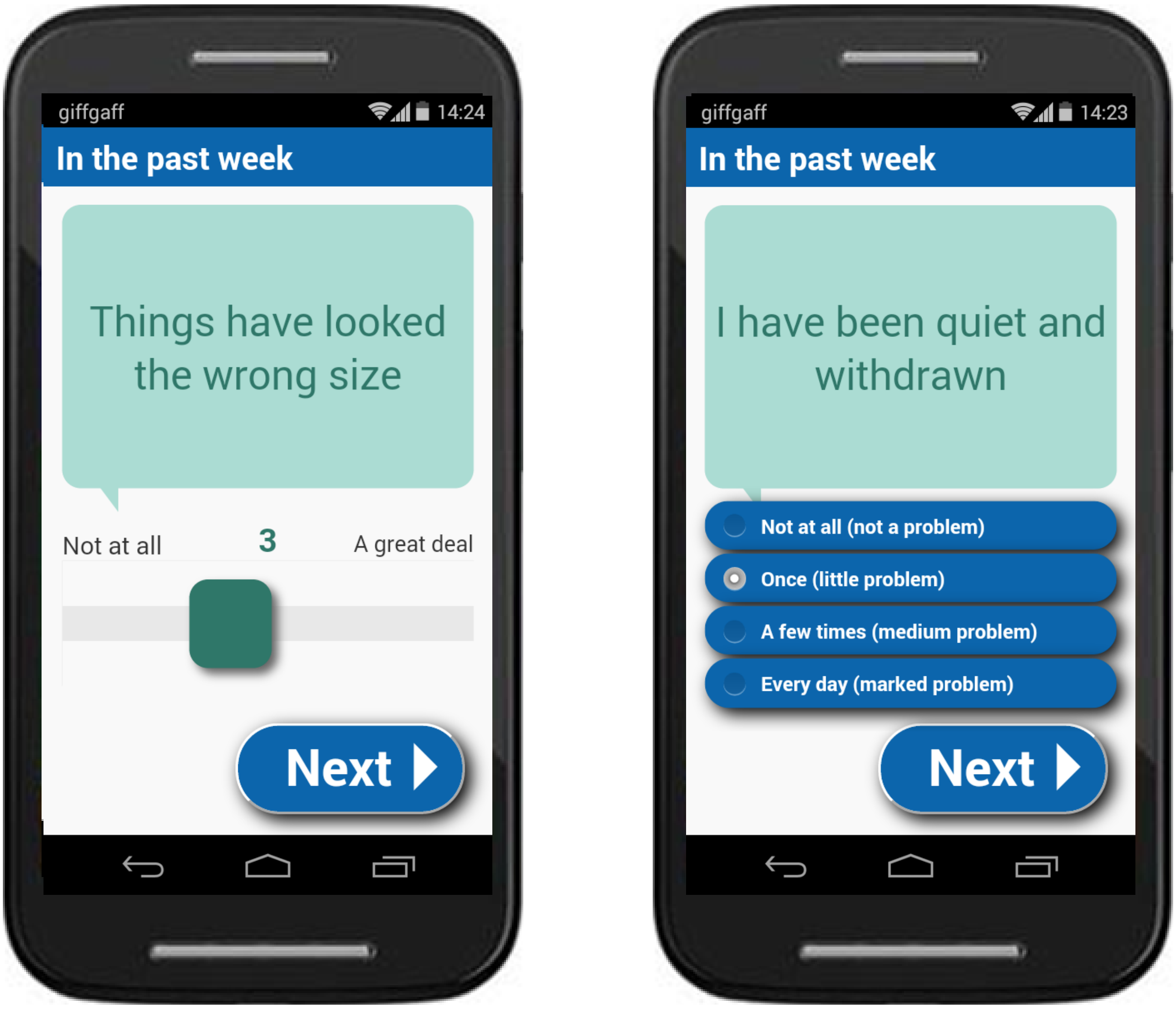

Supplementary Fig. 2: procedure for assessing primary relapse definition

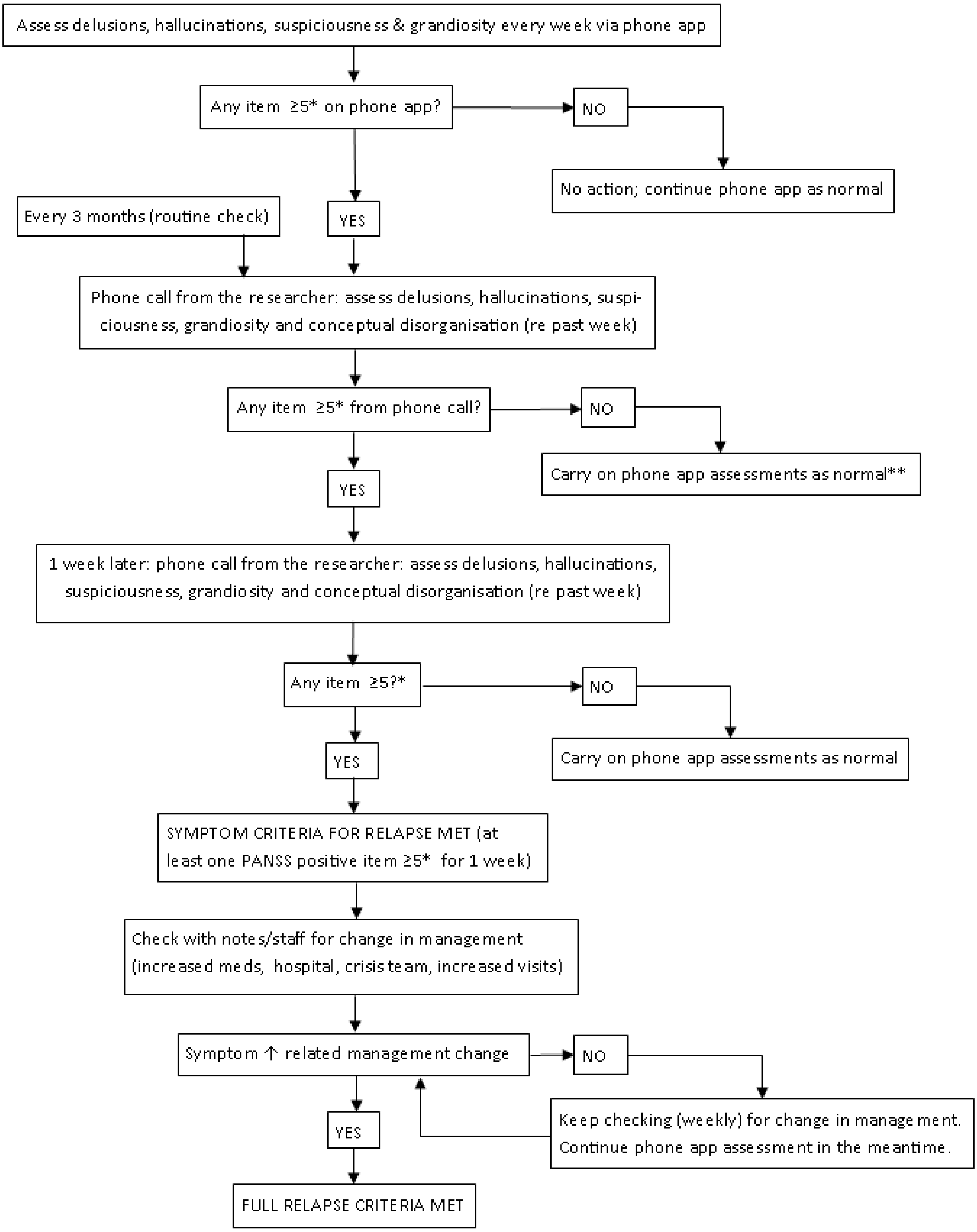

**Symptom increase criteria:*

Non-remitted individuals with all baseline Positive and Negative Syndrome Scale (PANSS) items <5: increase to ≥5 on any item.
Non-remitted with one or more baseline PANSS items ≥5: increase of 1 or more any PANSS positive item is required.
Remitted individuals: an increase to ≥4 on any PANSS positive item or an increase of 2 points, whichever is higher

Recalibration of threshold for receiving a phone call:

If app-reported symptoms prompted a phone call two weeks in a row but the phone call assessment showed no increase in symptoms, the threshold for receiving a phone call was adjusted, with the new threshold being one point above than the average of the previous four app reports.

Supplementary Fig. 3: App completion across the 24 hour response window (Wednesday 1.30pm to Thursday 1.30pm); distance from the centre is the number of completions in a 30 minute bin for that time, on a logarithmic scale.

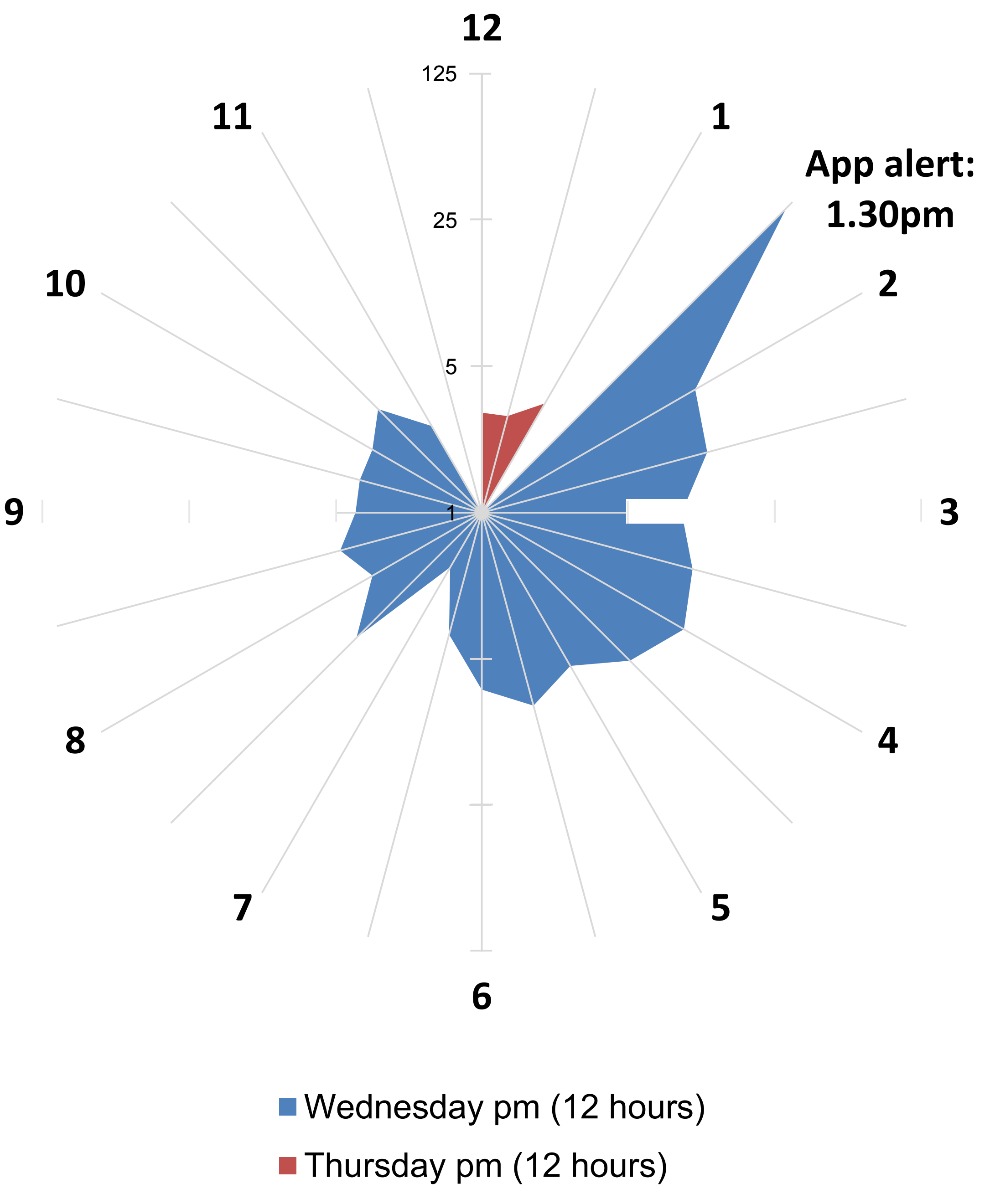

Supplementary Fig. 4: Average app use during the 6 month study period

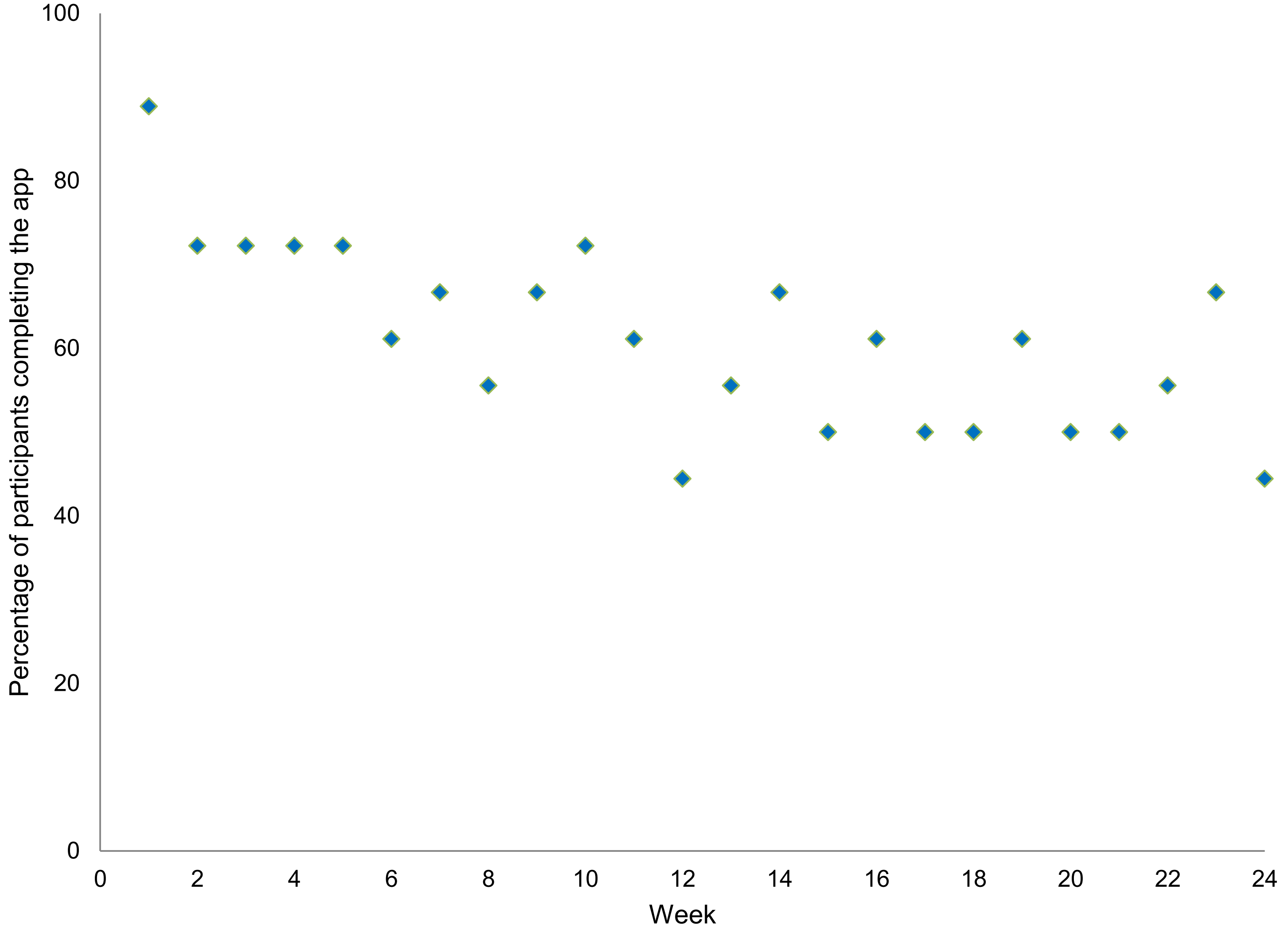

Supplementary Textbox 1: Details of symptom course and management changes in participants meeting full or partial relapse criteria

Participant 207

This participant did not meet remission criteria at baseline or during follow-up and had several baseline PANSS positive items scoring ≥5. Therefore the symptom increase criterion was: an increase of ≥1 point on any PANSS positive item. The threshold for receiving a PANSS phone interview was equivalent to the symptom increase criterion initially but this was exceeded two weeks in a row without any increase apparent in the resultant phone call, so was recalibrated to one point above the average of the previous 4 weeks' app-reported symptoms.

This participant met primary relapse criteria at week-15 of the study. He did not complete the app the week before relapse but informally reported an increase in his symptoms during the standard week-16 phone call; a PANSS positive phone interview confirmed an increase in hallucinations, with the hallucinations item score increasing from 5 to 6 (symptom increase criterion). The participant also reported that he had been admitted to the crisis team, which casenotes confirmed (management change criterion). The PANSS positive phone interview was repeated one week later to confirm that the symptom increase had lasted at least one week (duration criterion).

Casenotes reported a symptom increase from week-12 to week-19, and a crisis team admission beginning at week-15, so secondary relapse criteria were met.

Participant 230

This participant did not meet remission criteria at baseline or during follow-up and all baseline PANSS positive items were <5. Therefore the symptom increase criterion was: at least one item ≥5. The threshold for receiving a PANSS phone interview was equivalent to the symptom increase criterion throughout follow-up.

This individual met primary relapse criteria at week-6. Although he had filled in the app at week-6 and reported a slight increase in psychotic symptoms, this had not exceeded the threshold for a phone call interview. He subsequently missed two weeks of app reports but during the standard week-8 phone call reported that he had recently experienced a symptom increase. He declined to complete a phone PANSS interview at that point as he did not feel well enough. However, the interview was eventually completed (retrospectively) it confirmed an increase in hallucinations and suspiciousness scores from 3 to 5 (symptom increase criterion), beginning at week-8 and lasting 8 weeks in total (duration criterion). During the week-8 phone call, the participant mentioned that his medication had been increased, which casenotes confirmed (management change criterion).

Casenotes reported a symptom increase lasting from week-10 to week-18, with a medication increase at week-11, so secondary relapse criteria were met.

Participant 224

This individual did not meet remission criteria at baseline or during follow-up and had several baseline PANSS positive items scoring ≥5. Therefore the symptom increase criterion was: an increase of ≥1 point on any PANSS positive item. The threshold for receiving a PANSS phone interview was initially equivalent to the symptom increase criterion but this was exceeded two weeks in a row without any increase apparent in the resultant phone call, so it was recalibrated to one point above the average of the previous four weeks' app-reported symptoms.

This participant had very high levels of symptoms which were not adequately controlled by medication. Although he met secondary relapse criteria at week-19, this appears to be a false positive. At week-19 he met a new care co-ordinator for the first time, who reported a slight symptom increase which would not normally have resulted in a management change; however, given his high levels of residual symptoms, his (new) psychiatrist responded by changing his medication when she saw him at week-23.

Participant 227

This individual did not meet remission criteria at baseline or during follow-up and had several baseline PANSS positive items scoring ≥5. The symptom increase criterion was: an increase of ≥1 point on any PANSS positive item. The threshold for receiving a PANSS phone interview was equivalent to the symptom increase criterion throughout follow-up.

This participant's app-reported symptoms increased above threshold at week-11, prompting a PANSS phone interview which confirmed that delusions had increased from a score of 4 to 5. A phone call the following week confirmed that delusions remained elevated. However, despite meeting symptom increase and duration criteria, this client did not meet the management change criterion. It is possible that contextual factors precluded such a change: he lived in supported accommodation, was under the care of an extremely overstretched community mental health team and his care co-ordinator was on leave during the symptom increase. On the other hand the symptom increase may not have been severe enough to warrant intervention or may have been self-limiting (it appeared to have been triggered by an acute stressor which subsequently resolved).

Supplementary Fig. 5: pattern of conventional early signs, basic symptoms and psychotic symptoms in those meeting full or partial relapse definitions

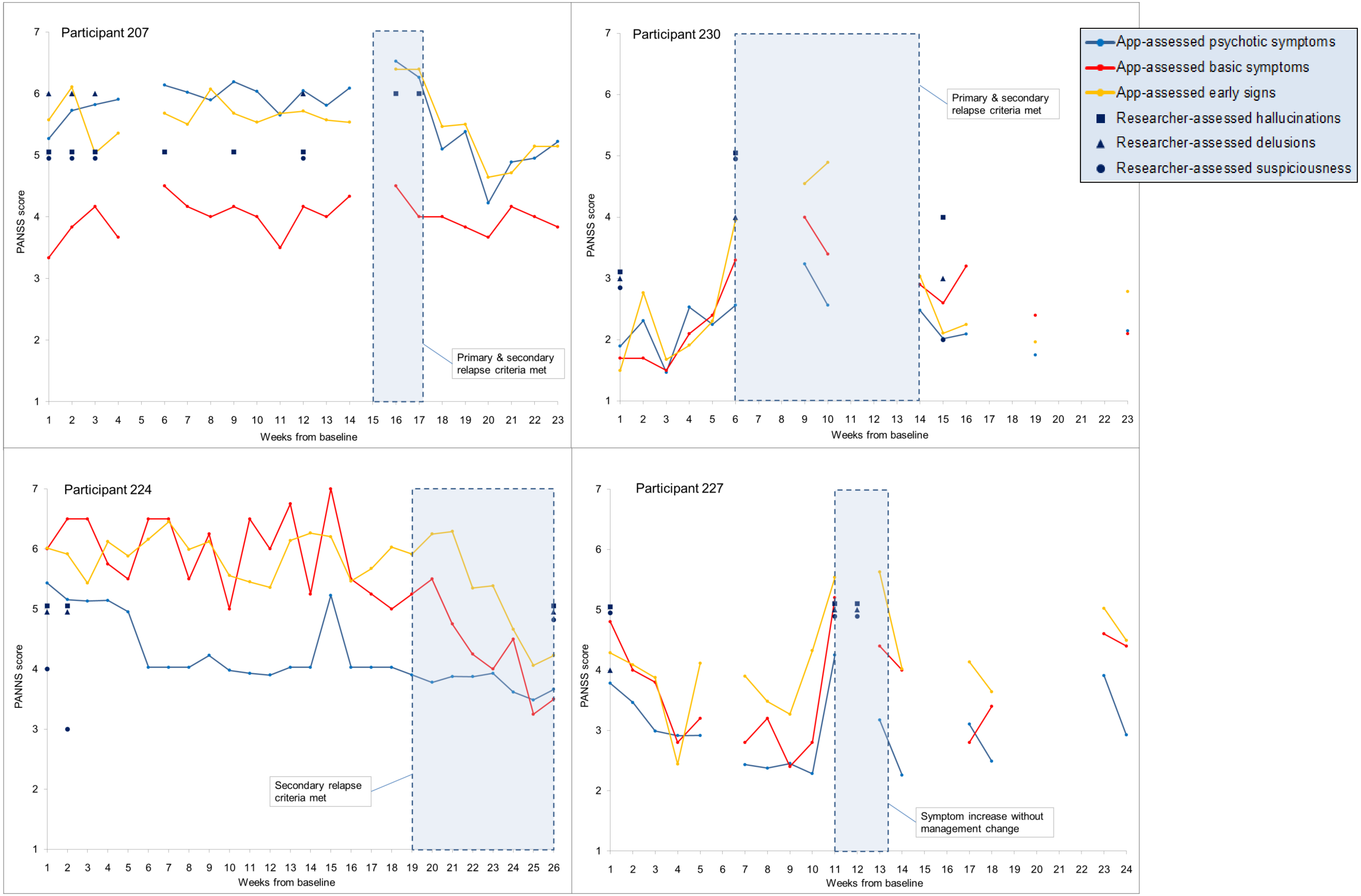

Supplementary Table 1: Framework matrix of qualitative interviews about study procedures

	**Reasons for taking part**	**Highlights**	**Lowlights**	**Researcher support**	**Phone calls**	**Financial reimbursement for phone calls**	**Other**
P205	The idea of the app appealed and may be able to help others, for example by decreasing duration of untreated psychosis: "Well I thought if there was an app available that would help people identify if they had psychosis that would be fantastic… cos, for some people the period between identifying is so long, it can go on and on and on and they can get worse and worse"	Normalisation of taking part in a study about psychosis and feeling that it could help people: "It's just a normalisation of well we have this psychosis but instead of looking at it in a negative way, look at it in a positive way, you could help people in the future, and I could be one of the service users of the app in the future as well"	None	Enough support	Phone calls "fine", including interviews.	No need for financial reimbursement for her personally. For others it would depend on the individual: "I think it depends on anybody's financial situation… I didn't mind cos I don't use all my minutes anyway...I wasn't in financial hardship, I was working, so it's fine. I think it'd depend on the individual"	None
P206	"I look at it as a fun. Like I said earlier on, and, to keep me going, to keep me busy"<br><br>"It's not about money… it will be useful for some people. We had a lot of people who donate their body when they die, they donate for people to study so if this is just one thing I can give for people to study then I'm happy with it"	"The questions, I love those questions!"	None	Texts helpful; enough support	Phone call during symptom increase was helpful and reassuring: "I believe, when I responded to the app… she knows something is happening to me and that's why you rang me… I really appreciate your call"<br><br>Phone calls encouraged him to continue with the app: "I really appreciate your call... I really liked it. Your call, your reminder, every time… because, you know, when you are doing something… I kind of feel I need somebody beside me just to cheer me [on] to do something."<br><br>Helped him feel a sense of belonging to the study: "yeah it keep me set on belonging. At least I'm taking part in something."	No need for financial reimbursement because "it's interesting, I have interest in taking part in it. I don't expect anything from it"	Practicing during app setup session was helpful: "we tried, we practiced it here the first day"
P207	"I just thought it might be interesting"	None	None	Texts helpful	Phone calls "absolutely fine", including interviews.	No need for financial reimbursement as "they're not really inconvenient or anything".	None
P208	"The thing that first attracted me to it was the shopping vouchers, I will admit… and then after the first couple of meetings I thought 'this is allright, this, it's a break from the norm, something else to do that's a little bit different'. And I quite enjoyed doing it. The whole lot."	"I've *thoroughly* enjoyed doing all of it really"	Length and personal nature of baseline interview questions off-putting to start with but ok once got used to it: "I think at first the questions that were asked about me being ill… I thought 'oh', sometimes they're not always easy to answer, but when get comfortable with somebody d'you know what I mean, and you start trusting them a bit, they're a lot easier to answer after that"	Enough support	"I liked the phone calls… it was good, to keep in contact".<br><br>Frequency of phone calls was "fine, yeah definitely"	Financial reimbursement: "I don't know really. I suppose it depends how long they are… who you're talking to, whether they're not so well at the time, I don't know. I suppose it's a bit of an incentive for somebody in't it really".<br><br>Thought that £5 per call was too much.	Possible bugs in the app - questions sometimes repeated; one week questions didn't come up: "one week it missed and it didn't come on at all… it just, I didn't get any questions at all. I'm thinking 'where's me questions?!'"<br><br>Financial reimbursement plenty; if anything too much phone credit: "I don't think it should have been more at all… If anything I think the phone credit was a bit much"

P209	Wanted to be altruistic but when it came to it he couldn't do it (dropped out of the study): "Doing the app and all that, trying to help people, it goes against my nature… I have trouble with anything like this…I don't know. I wish I could do things for other people. You know, help the university"<br><br>Something to do: "Trying to push myself… to basically stop sitting on the settee"<br><br>Might help him: "I thought it'd make me a little bit better, you know, at answering the phone and things like that"	None	Reasons for dropout:<br>"It was using the app. It's something new, I'm no good at that"<br>"I don't like having other people's phones in my house. In case someone picks it up or it gets dropped or anything like that"	Enough support	Phone calls fine as had met me already: "I don't mind, cos I'd seen you"	Not asked	The baseline interview was fine.
P211	"I don't mind, as long as - it may help understand the illness better… the university or the doctors, they might understand the illness better"	"It opened my eyes up on my illness a little bit better."	None	Texts helpful; enough support	Phone calls "good yeah. It's no problem", including interview	Financial reimbursement not necessary: "even if you did the interview for free it'd be allright"	None
P214	Don't remember	Sense of achievement: "When I got through it… it made me feel a bit better… I achieved something"	None	Texts helpful; enough support	Phone calls "fine", including interviews	Financial reimbursement not essential: "It doesn't bother me whether you get money or not. D'you know what I mean. I just feel that I've tried something new, d'you know what I mean".<br><br>However, "it's always nice to get money".	Pleased with herself that she did the study: "we have done it and I was quite impressed that we did it"
P215	"I got natural curiosity and I like the science, as I told you, so, if, before [researcher] proposed me that you know app so I say "why not!"…so gave it a try"	Enjoyed the first week when it was new: "just new maybe you know first week you know first er, er, first app where, first you know test when I take I'm bit enjoyed a bit you know"	Lack of insight so didn't think it was applicable to him, so felt like an "experimental rabbit"<br><br>Repeating questions: "that bit pissed me off er that you know the repeat questions"	"Definitely" enough support	Not asked	Not asked	None
P223	Help increase knowledge: "I thought you know um it would help you… knowing a bit more about it maybe"	None	"Probably just you know the same questions asked all the time if you know what I mean"	Enough support	Phone calls fine: "I didn't mind to be honest"	£5 for phone calls would be "allright"	None
P224	"[Care co-ordinator] just said to me like it's a new app and he said you just see you're feeling and you just answer a few question, and I said ok"	"I thought it was a nice app"<br><br>"I just like contributing something really…and my support worker will see it too so, he didn't feel the need then to ask me as many questions"	None	Enough support; felt ok to contact researcher when the charger broke: "I told her I broke the charger [laughing] like she wasn't angry or nothing she was, she text back and said it was fine, it's ok like so, it was nice yeah"	"Like she'd ring to check up sometimes and she'd ask how you were like"	Not being financially reimbursed for the phone calls "wasn't really a big deal to me"	This participant did not know about the graphs in the app. Improve training session to emphasise graphs more.
P225	Don't remember	None	Lack of literacy was the reason for dropout: "Basically I'm not very good at reading and spelling…that's one of the re-, well, that's the reason really"	Not asked	Phone calls "allright".	Would do the phone calls anyway but financial reimbursement "is a bonus".	Baseline interview was "a bit stressful at first, cos, you know, meeting a stranger and questions and stuff like but yeah it's allright, yeah"

P227	"I felt it was interesting…it intrigued me. Erm, I've never been part of a study like this before and it got my attention straight away thinking well… if it can help people in the future understand a little bit better about what we go through.. it's got to be a good thing really hasn't it"	Not asked	None	Text helpful; enough support	Phone calls "fine", though more difficult to talk about it if he'd had a bad week.	Financial reimbursement "an incentive"	No need for written instructions on how to use the app because "it was so easy… it was just a matter of pressing buttons, sliding it across".<br><br>However, this participant did not know about the graphs in the app. Improve training session to emphasise graphs more.
P230	Wanted to try out the app: "I wanted to try it out… and see how it – you know. Cos [researcher] showed me all the questions anyway she went through the phone with me …so it was quite easy"	"I did enjoy answering the questions"	Not asked	Enough support	During qualitative interview, participant reported that phone calls were "fine".<br><br>However, it was noted in the study feasibility diary that this participant was quite anxious about phone calls at some points. He declined to do PANSS interviews over the phone when he was unwell. When he was feeling better, he completed the phone interview but needed a lot of reassurance that his answers would be kept confidential.	Financial reimbursement helpful because sometimes phone calls took a long time for him: "yeah because, don't forget, on Friday it took over an hour didn't it".	This participant did not know about the graphs in the app. Improve training session to emphasise graphs more. Written instructions might be helpful.
P235	"I thought well you know I'll do five minutes a week, it's nowt is it, so I thought 'right'"; "I'm glad I could help"	None	None	Enough support	Phone calls "fine"	Financial reimbursement is "a good thing I spose. If it's er, funded by the NHS and it doesn't come out of your pocket, yeah"	None
P236	"If my… data goes towards you know helping people with you know similar er conditions… I'm happy to give five minutes of my time to, not only sort of assist me but if it's data that's going to create something you know useful for other people as well"<br><br>"I've done voluntary work for a few different places now, so…I suppose it's kind of giving a little bit back to… cos I've been helped quite a lot by the health services, I just thought if there's anything I can do to assist in making the services better or… yeah that's just summat I'd do"	"It's the satisfaction I suppose… that people could be grateful for you doing it"	None	Enough support	"I didn't really mind. It's like a procedure, procedural thing that you know was for my benefit more than anything so, that much I c-, I understand so… I know other people might find it a bit, might not understand, er… I dunno. I didn't find it intrusive or anything like that so…"	"Don't get me wrong. I don't mind doing it, you know, and… [pause]… I dunno. I suppose I, I'm not really doing it for the payment [laughing slightly] I suppose. That's just like an extra sort of"	None
P239	Previous positive experience of research	"Just that it was easy to do really. Even when things were not so good for me, it's still easy to answer it"	None	Enough support	Nice to have someone to chat to: "it was nice that yous was there to ring and just have a chat, cos then you felt like, cos I felt like the information I'm giving is going to yous, so it's nice to hear a voice isn't it?"<br><br>However, "sometimes when I feel really low I just want to be left alone and not mithered"	Some people might prefer not to be paid: "some people would say 'well, it's research so you shouldn't really have to pay' and some people feel like charity but some people also think they do need the money"<br><br>On the other hand it could be an incentive for some: "well, people're [laughing] more likely to do summat when they're getting paid for it aren't they?"	A lot of info in first session (interview and app setup combined for this participant) so hard to remember everything: "I can't remember if [researcher] told me that – there was that much that we was talking about it's just keeping in mind of everything isn't it?"
